# The Role of the Auditory Brainstem in Processing Musically Relevant Pitch

**DOI:** 10.3389/fpsyg.2013.00264

**Published:** 2013-05-13

**Authors:** Gavin M. Bidelman

**Affiliations:** ^1^Institute for Intelligent Systems, University of MemphisMemphis, TN, USA; ^2^School of Communication Sciences and Disorders, University of MemphisMemphis, TN, USA

**Keywords:** musical pitch perception, consonance and dissonance, tonality, auditory event-related potentials, brainstem response, frequency-following response (FFR), musical training, auditory nerve

## Abstract

Neuroimaging work has shed light on the cerebral architecture involved in processing the melodic and harmonic aspects of music. Here, recent evidence is reviewed illustrating that subcortical auditory structures contribute to the early formation and processing of musically relevant pitch. Electrophysiological recordings from the human brainstem and population responses from the auditory nerve reveal that nascent features of tonal music (e.g., consonance/dissonance, pitch salience, harmonic sonority) are evident at early, subcortical levels of the auditory pathway. The salience and harmonicity of brainstem activity is strongly correlated with listeners’ perceptual preferences and perceived consonance for the tonal relationships of music. Moreover, the hierarchical ordering of pitch intervals/chords described by the Western music practice and their perceptual consonance is well-predicted by the salience with which pitch combinations are encoded in subcortical auditory structures. While the neural correlates of consonance can be tuned and exaggerated with musical training, they persist even in the absence of musicianship or long-term enculturation. As such, it is posited that the structural foundations of musical pitch might result from innate processing performed by the central auditory system. A neurobiological predisposition for consonant, pleasant sounding pitch relationships may be one reason why these pitch combinations have been favored by composers and listeners for centuries. It is suggested that important perceptual dimensions of music emerge well before the auditory signal reaches cerebral cortex and prior to attentional engagement. While cortical mechanisms are no doubt critical to the perception, production, and enjoyment of music, the contribution of subcortical structures implicates a more integrated, hierarchically organized network underlying music processing within the brain.

In Western tonal music, the octave is divided into 12 equally spaced pitch classes (i.e., semitones). These elements can be further arranged into seven tone subsets to construct the diatonic major/minor scales that define tonality and musical key. Music theory and composition stipulate that the pitch combinations (i.e., intervals) formed by these scale-tones carry different weight, or importance, within a musical framework (Aldwell and Schachter, 2003). That is, pitch intervals follow a hierarchical organization in accordance with their functional role in musical composition (Krumhansl, 1990). Intervals associated with stability and finality are regarded as *consonant* while those associated with instability (i.e., requiring resolution) are regarded as *dissonant*. Given their anchor-like function in musical contexts, it is perhaps unsurprising that consonant pitch relationships occur more frequently in tonal music than dissonant relationships (Budge, 1943; Vos and Troost, 1989). Ultimately, it is the ebb and flow between consonance and dissonance which conveys musical tension and establishes the structural foundations of melody and harmony, the fundamental building blocks of Western tonal music (Rameau, 1722/1971; Krumhansl, 1990).

## The Perception of Musical Pitch: Sensory Consonance and Dissonance

**Figure 1:**
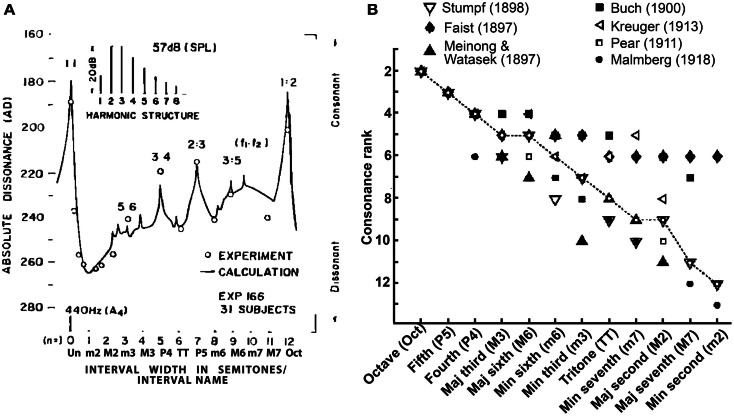
**Consonance rankings for chromatic scale tone combinations of Western music practice**. **(A)** Consonance (i.e., “pleasantness”) ratings reported by Kameoka and Kuriyagawa (1969b) for two-tone intervals (dyads). Stimuli were composed of two simultaneously sounding complex tones (inset). The spacing between fundamental frequencies (*f*_1_, *f*_2_) was varied to form the various chromatic intervals within the range of an octave; the lower tone (*f*_1_) was always fixed at 440 Hz and the upper tone (*f*_2_) varied from 440 to 880 Hz in semitone spacing. Note the higher behavioral ratings for the consonant pitch relationships [e.g., 0 (Un), 7 (P5), 12 (Oct) semitones] relative to dissonant relationships [e.g., 2 (m2), 6 (TT), 11 (M7) semitones] as well as the hierarchical arrangement of intervals (Un > Oct > P5 > P4 > M6, etc). **(B)** Rank order of musical interval consonance ratings reported across seven psychophysical studies (Faist, 1897; Meinong and Witasek, 1897; Buch, 1900; Pear, 1911; Kreuger, 1913; Malmberg, 1918; Stumpf, 1989). Open circles represent the median consonance rank assigned to each of the 12 chromatic dyads. Figures adapted from Kameoka and Kuriyagawa (1969b) and Schwartz et al. (2003) with permission from The Acoustical Society of America and Society for Neuroscience, respectively.

The music cognition literature distinguishes the aforementioned *musical* definitions from those used to describe the *psychological* attributes of musical pitch. The term *tonal-* or *sensory-consonance-dissonance* refers to the perceptual quality of two or more simultaneous tones presented in isolation (Krumhansl, 1990) and is distinct from consonance arising from contextual or cognitive influences (see Dowling and Harwood, 1986, for a discussion of non-sensory factors). Perceptually, consonant pitch relationships are described as sounding more pleasant, euphonious, and beautiful than dissonant combinations which sound unpleasant, discordant, or rough (Plomp and Levelt, 1965). Consonance is often described parsimoniously as the absence of dissonance. A myriad of empirical studies have quantified the perceptual qualities of musical pitch relationships. In such behavioral experiments, listeners are typically played various two-tone pitch combinations (dyads) constructed from the musical scale and asked to rate their degree of consonance (i.e., “pleasantness”). Examples of such ratings, as reported in the seminal studies of Kameoka and Kuriyagawa (1969a,b), are shown in Figure 1A. The rank order of intervals according to their perceived consonance is shown in Figure 1B. Two trends emerge from the pattern of ratings across a number of studies: (i) listeners routinely prefer consonant pitch relationships (e.g., octave, fifth, fourth, etc.) to their dissonant counterparts (e.g., major/minor second, sevenths) and (ii) intervals are not heard in a strict binary manner (i.e., consonant vs. dissonant) but rather, are processed differentially based on their degree of perceptual consonance (e.g., Kameoka and Kuriyagawa, 1969a,b; Krumhansl, 1990). These behavioral studies demonstrate that musical pitch relationships are perceived *hierarchically* and in an arrangement that parallels their relative use and importance in music composition (Krumhansl, 1990; Schwartz et al., 2003).

Interestingly, the preference for consonance and the hierarchical nature of musical pitch perception is reported even for non-musician listeners (Van De Geer et al., 1962; Tufts et al., 2005; Bidelman and Krishnan, 2009). Thus, while the perceptual nuances of music might be augmented with experience (McDermott et al., 2010; Bidelman et al., 2011c) – or degraded with impairments (e.g., amusia: Cousineau et al., 2012) – a perceptual bias for consonant pitch combinations persists even in the absence of musical training. Indeed, this bias for consonance emerges early in life, well before an infant is exposed to the stylistic norms of culturally specific music (Trehub and Hannon, 2006). Evidence from animal studies indicates that even non-human species (e.g., sparrows and Japanese monkeys) discriminate consonant from dissonant pitch relationships (Izumi, 2000; Watanabe et al., 2005; Brooks and Cook, 2010) and some even show musical preferences similar to human listeners (e.g., Bach > Schönberg) (Sugimoto et al., 2010). These data provide convincing evidence that certain aspects of music perception might be innate, a byproduct of basic properties of the auditory system.

The current review aims to provide a comprehensive overview of recent work examining the psychophysiological bases of consonance, dissonance, and the hierarchical foundations of musical pitch. Discussions of these musical phenomena have enjoyed a rich history of arguments developed over many centuries. As such, treatments of early explanations are first provided based on mathematical, acoustic, and psychophysical accounts implicating peripheral auditory mechanisms (e.g., cochlear mechanics) in musical pitch listening. Counterexamples are then provided which suggest that strict acoustic and cochlear theories are inadequate to account for the findings of recent studies examining human consonance judgments. Lastly, recent neuroimaging evidence is highlighted which supports the notion that the perceptual attributes of musical pitch are rooted in *neurophysiological* processing performed by the central nervous system. Particular attention is paid to recent studies examining the neural encoding of musical pitch using scalp-recorded brainstem responses elicited from human listeners. Brainstem evoked potentials demonstrate that the perceptual correlates of musical consonance and pitch hierarchy are well represented in subcortical auditory structures, suggesting that attributes important to music listening emerge well before the auditory signal reaches cerebral cortex. The contribution of subcortical mechanisms implies that music engages a more integrated, hierarchically organized network tapping both sensory (pre-attentive) and cognitive levels of brain processing.

## Historical Theories and Explanations for Musical Consonance and Dissonance

### The acoustics of musical consonance

Early explanations of consonance and dissonance focused on the underlying acoustic properties of musical intervals. It was recognized as early as the ancient Greeks, and later by Galilei (1638/1963), that pleasant sounding (i.e., consonant) musical intervals were formed when two vibrating entities were combined whose frequencies formed simple integer ratios (e.g., 3:2 = perfect fifth, 2:1 = octave). In contrast, “harsh” or “discordant” (i.e., dissonant) intervals were created by combining tones with complex ratios (e.g., 16:15 = minor second). By these purely mathematical standards, consonant intervals were regarded as divine acoustic relationships superior to their dissonant counterparts and, as a result, were heavily exploited by early composers (for a historic account, see Tenney, 1988). Indeed, the most important pitch relationships in music, including the major chord, can be derived directly from the first few components of the harmonic series (Gill and Purves, 2009). Yet, while attractive *prima facie*, the long held theory that the ear prefers simple ratios is no longer tenable when dealing with contemporary musical tuning systems. For example, the ratio of the consonant perfect fifth under modern equal temperament (442:295) is hardly a small integer relationship. Though intimately linked, explanations of consonance-dissonance based purely on these physical constructs (e.g., frequency ratios) are, in and of themselves, insufficient in describing all of the cognitive aspects of musical pitch (Cook and Fujisawa, 2006; Bidelman and Krishnan, 2009). Indeed, it is possible for an interval to be esthetically dissonant while mathematically consonant, or vice versa (Cazden, 1958, p. 205). For example, tones combined at simple ratios (traditionally considered consonant), can be judged to be dissonant when their frequency components are stretched (i.e., made inharmonic) from their usual position in the harmonic series (Slaymaker, 1970) or when occurring in an unexpected musical context (Dowling and Harwood, 1986). These experimental paradigms cleverly disentangle stimulus acoustics (e.g., frequency ratios) from behavioral consonance judgments and, in doing so, indicate that pure acoustic explanations are largely inadequate as a sole basis of musical consonance.

### Psychophysiology of musical consonance

#### Psychophysical roughness/beating and the cochlear critical band

Helmholtz (1877/1954) offered some of the earliest psychophysical explanations for sensory consonance-dissonance. He observed that when adjacent harmonics in complex tones interfere they create the perception of “roughness” or “beating,” percepts closely related to the perceived dissonance of tones (Terhardt, 1974). Consonance, on the other hand, occurs in the absence of beating, when low-order harmonics are spaced sufficiently far apart so as not to interact. Empirical studies suggest this phenomenon is related to cochlear mechanics and the critical-band hypothesis (Plomp and Levelt, 1965). This theory postulates that the overall consonance-dissonance of a musical interval depends on the total interaction of frequency components within single auditory filters. Pitches of consonant dyads have fewer partials which pass through the same critical bands and therefore, yield more pleasant percepts; in contrast, the partials of dissonant intervals compete within individual channels and as such, yield discordant percepts.

Unfortunately, roughness/beating is often difficult to isolate from consonance percepts given that both covary with the spacing between frequency components in the acoustic waveform, and are thus, intrinsically coupled. While within-channel interactions may produce some amount of dissonance, modern empirical evidence indicates that beating/roughness plays only a minor role in its perception. Indeed, at least three pieces of evidence support the notion that consonance may not be mediated by roughness/beating, *per se*. First, psychoacoustic findings indicate that roughness percepts are dominated by lower modulation rates (∼30–150 Hz) (Terhardt, 1974; McKinney et al., 2001, p. 2). Yet, highly dissonant intervals are heard for tones spaced well beyond this range (Bidelman and Krishnan, 2009; McDermott et al., 2010). Second, dichotic listening tasks can been used to eliminate the monaural interactions necessary for roughness and beating. In these experiments, the constituent notes of a musical interval are separated between the ears. Dichotic listening ensures that roughness/beating along the cochlear partition is eliminated, as each ear processes a perfectly periodic, singular tone. Nevertheless, dichotic presentation does not alter human consonance judgments (Houtsma and Goldstein, 1972; Bidelman and Krishnan, 2009; McDermott et al., 2010), indicating that cochlear interactions (and the critical band) are insufficient explanations for explaining consonance/dissonance percepts. Lastly, lesion studies indicate a dissociation between roughness and the perception of dissonance as one percept can be selectively impaired independently of the other (Tramo et al., 2001). Taken together, converging evidence suggests that roughness/beating may not be as important a factor in sensory consonance-dissonance as conventionally thought (e.g., Helmholtz, 1877/1954; Plomp and Levelt, 1965; Terhardt, 1974).

#### Tonal fusion and harmonicity

Alternate theories have suggested musical consonance is determined by the sense of “fusion” or “tonal affinity” between simultaneously sounding pitches (Stumpf, 1890). Pitch fusion describes the degree to which multiple pitches are heard as a single, unitary tone (DeWitt and Crowder, 1987). Fusion is closely related to harmonicity, which describes how well a sound’s acoustic spectrum agrees with a single harmonic series (Gill and Purves, 2009; McDermott et al., 2010; Bidelman and Heinz, 2011). Pitch relationships with more coinciding partials have spectra that are more harmonic (e.g., octave, perfect fifth). As a result, they are heard as being fused which consequently creates the sensation of consonance. In contrast, pitch relationships which are more inharmonic (e.g., minor second, tritone) have spectra which diverge from a single harmonic series, are less fused perceptually, and create the quality of dissonance. Under this hypothesis then, the auditory system formulates consonance based on the harmonicity of sound. Support for the fusion/harmonicity premise stems from experiments examining inharmonic tone complexes, which show that consonance is obtained when tones share coincident partials, even when other factors known to influence consonance are varied, e.g., the ratio of note fundamental frequencies or roughness/beating (Slaymaker, 1970; Bidelman and Krishnan, 2009; McDermott et al., 2010; Bidelman and Heinz, 2011). For example, even a complex ratio (typically associated with dissonance) can be heard as consonant if it fits into the template of a single complex tone. Recent behavioral work supports the dominance of harmonicity in musical pitch percepts: consonance preferences are strongly correlated with a preference for harmonicity but not, for example, a preference for lack of roughness (McDermott et al., 2010).

#### Neurophysiology of musical consonance

The fact that these perceptual factors do not depend on long-term enculturation or musical training and have been reported even in non-human species (Izumi, 2000; Watanabe et al., 2005; Brooks and Cook, 2010; Sugimoto et al., 2010) suggests that the basis of musical consonance and pitch hierarchy might be rooted in the fundamental processing and/or constraints of the auditory system (Trehub and Hannon, 2006). In particular, the similarity in percepts under dichotic listening indicates that consonance must be computed centrally by deriving information from the combined signals relayed from both cochleae (Houtsma and Goldstein, 1972; Bidelman and Krishnan, 2009). Indeed, converging evidence suggests that these properties of musical pitch may be reflected in intrinsic, temporal firing patterns, and synchronization of auditory neurons (Boomsliter and Creel, 1961; Ebeling, 2008). Having ruled out pure mathematical, acoustical, and cochlear explanations, neurophysiological studies will now be examined which suggest a neural basis of musical consonance, dissonance, and tonal hierarchy.

## Neural Correlates of Consonance, Dissonance, and Musical Pitch Hierarchy

Neuroimaging methods have offered a window into the cerebral architecture underlying the perceptual attributes of musical pitch. Functional magnetic resonance imaging (fMRI), for example, has shown differential and enhanced activation across cortical regions (e.g., inferior/middle frontal gyri, premototor cortices, interior parietal lobule) when processing consonant vs. dissonant tonal relationships (Foss et al., 2007; Minati et al., 2009; Fujisawa and Cook, 2011). Scalp-recorded event-related brain potentials (ERPs) have proved to be a particularly useful technique to non-invasively probe the neural correlates of musical pitch. ERPs represent the time-locked neuroelectric activity of the brain generated by the activation of neuronal ensembles within cerebral cortex. The auditory cortical ERP consists of a series of voltage deflections (i.e., “waves”) within the first ∼250 ms after the onset of sound. Each deflection represents the subsequent activation in a series of early auditory cortical structures including thalamus and primary/secondary auditory cortex (Näätänen and Picton, 1987; Scherg et al., 1989; Picton et al., 1999). The millisecond temporal resolution of ERPs provides an ideal means to investigate the time-course of music processing within the brain not afforded by other, more sluggish neuroimaging methodologies (e.g., fMRI).

### Cortical correlates of musical consonance

**Figure 2:**
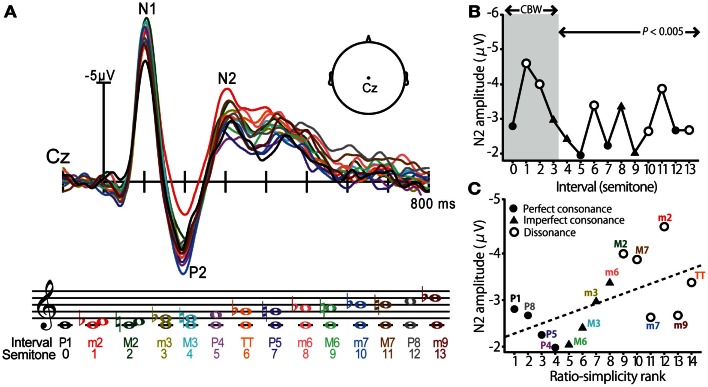
**Cortical event-related potentials (ERPs) elicited by musical dyads**. **(A)** Cortical ERP waveforms recorded at the vertex of the scalp (Cz lead) in response to chromatic musical intervals. Response trace color corresponds to the evoking stimulus denoted in music notation. Interval stimuli were composed of two simultaneously sounding pure tones. **(B)** Cortical N2 response magnitude is modulated by the degree of consonance; dissonant pitch relationships evoke larger N2 magnitude than consonant intervals. The shaded region demarcates the critical bandwidth (CBW); perceived dissonance created by intervals larger than the CBW cannot be attributed to cochlear interactions (e.g., beating between frequency components). Perfect consonant intervals (filled circles); imperfect consonant intervals (filled triangles); dissonant intervals (open circles) **(C)** Response magnitude is correlated with the degree of simplicity of musical pitch intervals; simpler, more consonant pitch relationships (e.g., P1, P8, P5) elicit smaller N2 than more complex, dissonant pitch relationships (e.g., M2, TT, M7). Figure adapted from Itoh et al. (2010) with permission from The Acoustical Society of America.

Using far-field recorded ERPs, neural correlates of consonance, dissonance, and musical scale pitch hierarchy have been identified at a cortical level of processing (Brattico et al., 2006; Krohn et al., 2007; Itoh et al., 2010). Cortical evoked responses elicited by musical intervals, as reported by (Itoh et al., 2010), are shown in Figure 2. In this experiment, listeners were played a random sequence of dyadic intervals (0–13 semitones) in a passive listening task while ERPs were recorded at the scalp. The use of pure tones ensured minimal roughness at the auditory periphery. Modulations in cortical activity were observed in the prominent waves of the ERP but were especially apparent in the later endogenous P2-N2 complex at a latency of ∼200–300 ms (Figure 2A). Indeed, N2 magnitude varied with the dyad’s degree of consonance; intervals established in previous studies as dissonant – those which are unpleasant to the ear – elicited larger N2 responses than the more pleasant sounding, consonant pitch intervals (Figure 2B). Importantly, these effects were observed even when the interval’s separation exceeded the critical bandwidth (∼3 semitones) suggesting that consonance, and its neural underpinnings, were computed based on properties other than roughness. Further examination revealed that N2 magnitude also corresponded with a measure of the intervals’ “ratio simplicity” (Schellenberg and Trehub, 1994), defined as 1/log(*X* + *Y*) for the ratio *X:Y* (Figure 2C). These results demonstrate that (i) cortical activity distinguishes pitch relationships according to their consonance and in a manner consistent with standard musical practice and (ii) the central auditory system exploits the harmonicity of sound to code the perceptual pleasantness of music. These studies clearly demonstrate that *cortical activity* is especially sensitive to the pitch relationships found in music. Yet, a natural question that emerges is whether these neural correlates emerge prior to the auditory cortices, e.g., at *subcortical* stages of auditory processing.

### Brainstem correlates of musical consonance and scale pitch hierarchy

To assess human subcortical auditory processing, electrophysiological studies have utilized the frequency-following responses (FFRs). The FFR is a sustained evoked potential characterized by a periodic waveform which follows the individual cycles of the stimulus (for review, see Krishnan, 2007; Chandrasekaran and Kraus, 2010; Skoe and Kraus, 2010). Based on its latency (Smith et al., 1975), lesion data (Smith et al., 1975; Sohmer et al., 1977), and known extent of phase-locking in the brainstem (Wallace et al., 2000; Aiken and Picton, 2008; Alkhoun et al., 2008), a number of studies recognize the inferior colliculus (IC) of the midbrain as the primary generator of the FFR. Employing this response, recent work from our lab has explored the neural encoding of musical pitch-relevant information at the level of the brainstem.

**Figure 3:**
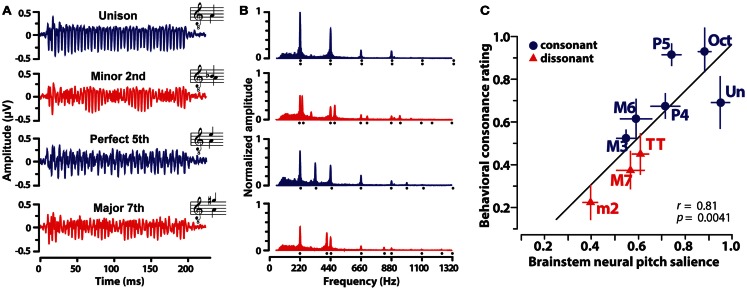
**Human brainstem frequency-following responses (FFRs) elicited by musical dyads**. Grand average FFR waveforms **(A)** and their corresponding frequency spectra **(B)** evoked by the dichotic presentation of four representative musical intervals. Consonant intervals, blue; dissonant intervals, red. **(A)** Clearer, more robust periodicity is observed for consonant relative to dissonant intervals. **(B)** Frequency spectra reveal that FFRs faithfully preserve the harmonic constituents of *both* musical notes of the interval (compare response spectrum, filled area, to stimulus spectrum, harmonic locations denoted by dots). Consonant intervals evoked more robust spectral magnitudes across harmonics than dissonant intervals. Amplitudes are normalized relative to the unison. **(C)** Correspondence between FFR pitch salience computed from brainstem responses and behavior consonance ratings. Neural responses well predict human preferences for musical intervals. Note the systematic clustering of consonant and dissonant intervals and the maximal separation of the unison (most consonant interval) from the minor second (most dissonant interval) in the neural-behavioral space. Data from Bidelman and Krishnan (2009).

In a recent study (Bidelman and Krishnan, 2009) recorded FFRs elicited by nine musical dyads that varied in their degree of consonance and dissonance. Dichotic stimulus presentation ensured that peripheral roughness/beating was minimized and that consonance percepts were computed centrally after binaural integration (Houtsma and Goldstein, 1972). In addition, only non-musicians were recruited to ensure participants had no explicit exposure to the rules of musical theory, a potential bias, or knowledge of learned labels for musical pitch relationships. Exemplar FFRs and response spectra evoked by a subset of the dyads are shown in Figure 3. From brainstem responses, a measure of “neural pitch salience” was computed using a harmonic sieve analysis (Cedolin and Delgutte, 2005) to quantify the harmonicity of the neural activity (see Bidelman and Krishnan, 2009 for details). Essentially, this algorithm is a time-domain analog of the classic pattern recognition model of pitch whereby a “central pitch processor” matches harmonic information contained in the response to an internal template in order to compute the heard pitch (Goldstein, 1973; Terhardt et al., 1982). Results showed that brainstem responses to consonant intervals were more robust and yielded stronger neural pitch salience than those to dissonant intervals. In addition, the ordering of neural salience across musical intervals followed the hierarchical arrangement of pitch stipulated by Western music theory (Rameau, 1722/1971; Krumhansl, 1990). Lastly, neural pitch salience was well-correlated with listeners’ behavioral consonance ratings (Figure 3C). That is, musical preferences could be predicted based on an individual’s underlying subcortical response activity. Subsequent studies showed that brainstem encoding could similarly predict the sonority ratings of more complex musical pitch relationships including the four most common triadic chords in music (Bidelman and Krishnan, 2011). Together, results suggest that in addition to cortical processing (e.g., Itoh et al., 2010), *subcortical* neural mechanisms (i) show preferential encoding of consonant musical relationships and (ii) preserve and predict the hierarchical arrangement of pitch as described in music practice and in psychophysical studies.

Importantly, these strong brain-behavior relationships have been observed in non-musician listeners and under conditions of passive listening (most subjects fell asleep during EEG testing). These factors imply that basic perceptual aspects of music might be rooted in intrinsic sensory processing. Unfortunately, these brainstem studies employed adult human listeners. As such, they could not rule out the possibility that non-musicians’ brain responses might have been preferentially tuned via long-term enculturation and/or implicit exposure to the norms of Western music practice.

### Auditory nerve correlates of musical consonance

To circumvent confounds of musical experience, enculturation, memory, and other top-down factors which influence the neural code for music, Bidelman and Heinz (2011) investigated whether the correlates of consonance were present at very initial stages of the auditory pathway. Auditory nerve (AN) fiber responses were simulated using a computational model of the auditory periphery (Zilany et al., 2009). This model – originally used to describe AN response properties in the cat – incorporates many of the most important properties observed in the peripheral auditory system including, cochlear filtering, level-dependent gain (i.e., compression) and bandwidth control, as well as two-tone suppression. Details of this phenomenological model are beyond the scope of the present review. Essentially, the model accepts a sound input (e.g., musical interval) and outputs a realistic train of action potentials (i.e., spikes) that accurately simulates the discharge pattern of single AN neurons as recorded in animal studies (Zilany and Bruce, 2006). Actual neurophysiological experiments are often plagued by limited recording time, stimuli, and small sample sizes so their conclusions are often restricted. Modeling thus allowed for the examination of (i) possible differential AN encoding across a large continuum (i.e., 100s) of musical and non-musical pitch intervals and (ii) activation across an array of nerve fibers spanning the entire cochlear partition.

**Figure 4:**
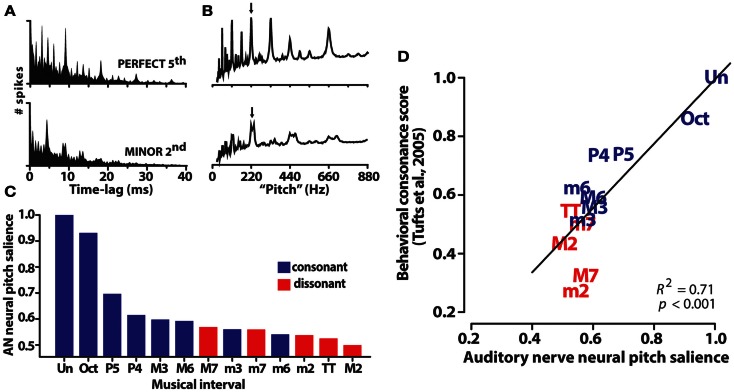
**Auditory nerve (AN) responses to musical dyads**. **(A)** Population level interspike interval histograms (ISIHs) for a representative consonant (perfect fifth: 220 + 330 Hz) and dissonant (minor second: 220 + 233 Hz) musical interval. ISIHs quantify the periodicity of spike discharges from a population of 70 AN fibers driven by a single two-tone musical interval. **(B)** Neural pitch salience profiles computed from ISIHs via harmonic sieve analyses quantify the salience of all possible pitches contained in AN responses based on harmonicity of the spike distribution. Their peak magnitude (arrows) represents a singular measure of neural pitch salience for the eliciting musical interval. **(C)** AN pitch salience across the chromatic intervals is more robust for consonant than dissonant intervals. Rank order of the intervals according to their neural pitch salience parallels the hierarchical arrangement of pitches according to Western music theory (i.e., Un > Oct > P5, >P4, etc.). **(D)** AN pitch representations predict the hierarchical order of behavioral consonance judgments of human listeners (behavioral data from normal-hearing listeners of Tufts et al., 2005). AN data reproduced from Bidelman and Heinz (2011).

Auditory nerve population responses were obtained by pooling single-unit responses from 70 fibers with characteristic frequencies spanning the range of human hearing. Spike trains were recorded in response to 220 dyads within the range of an octave where *f*_1_/*f*_2_ separation varied from the unison (i.e., *f*_2_ = *f*_1_) to the octave (i.e., *f*_2_ = 2*f*_1_). First-order interspike interval histograms computed from raw spike times allowed for the quantification of periodicity information contained in the aggregate AN response (Figure 4A). Adopting techniques of (Bidelman and Krishnan, 2009), harmonic sieve analysis was used to extract the salience of pitch-related information encoded in the entire AN ensemble. Neural pitch salience profiles elicited by exemplar consonant (P5) and dissonant (m2) musical dyads are shown in Figure 4B. The maximum of each profile provided a singular estimate of the neural salience for each dyad stimulus. Interestingly, rank order of the chromatic intervals according to this salience magnitude followed a predictable pattern; consonant intervals – those judged more pleasant sounding by listeners – yielded higher neural rankings than dissonant intervals (e.g., M7, TT, m2) (Figure 4C). Additionally, although neural rank ordering was derived from responses at the level of AN, they showed close agreement to rankings stipulated by Western music theory as well as those obtained from human listeners in psychophysical studies (e.g., Figure 1). As with human brainstem FFRs, AN responses were well-correlated with perceptual judgments of consonance (Figure 4D). That is, the hierarchical perception and perceived pleasantness of musical stimuli could be well-predicted based on neural responses at the level of AN. Our earlier findings from human brainstem ERPs suggested that such preferences might emerge based on subcortical neurocomputations well before cerebral cortex. Our AN modeling studies extend these results, and further suggest they might even be rooted in the most peripheral sites of the auditory brain.

In follow-up analyses, it was shown that neither acoustic nor traditional psychophysical explanations (e.g., periodicity, roughness/beating) could fully account for human consonance ratings (Bidelman and Heinz, 2011). Of the number of explanatory factors examined, neural harmonicity was the most successful predictor of human percepts (cf. Bidelman and Krishnan, 2009). Recent psychoacoustical evidence corroborates these findings and confirms that the perception of consonance-dissonance is governed primarily by the harmonicity of a musical interval/chord and not its roughness or beating (McDermott et al., 2010; Cousineau et al., 2012). That is, converging evidence indicates that consonance is largely computed based on the degree to which a stimulus sounds like a single harmonic series.

## The Hierarchical Nature and Basis of Subcortical Pitch Processing

**Figure 5:**
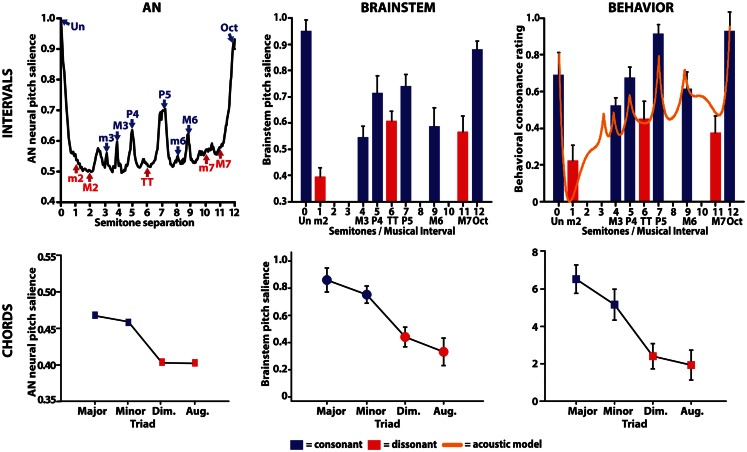
**Comparison between auditory nerve, human brainstem evoked potentials, and behavioral responses to musical intervals**. (Top left) AN responses correctly predict perceptual attributes of consonance, dissonance, and the hierarchical ordering of musical dyads. AN neural pitch salience is shown as a function of the number of semitones separating the interval’s lower and higher pitch over the span of an octave (i.e., 12 semitones). Consonant musical intervals (blue) tend to fall on or near peaks in neural pitch salience whereas dissonant intervals (red) tend to fall within trough regions, indicating more robust encoding for the former. Among intervals common to a single class (e.g., all consonant intervals), AN responses show differential encoding resulting in the hierarchical arrangement of pitch typically described by Western music theory (i.e., Un > Oct > P5, >P4, etc.). (Top middle) neural correlates of musical consonance observed in human brainstem responses. As in the AN, brainstem responses reveal stronger encoding of consonant relative to dissonant pitch relationships. (Top right) behavioral consonance ratings reported by human listeners. Dyads considered consonant according to music theory are preferred over those considered dissonant [minor second (m2), tritone (TT), major seventh (M7)]. For comparison, the solid line shows predictions from a mathematical model of consonance and dissonance (Sethares, 1993) where local maxima denote higher degrees of consonance than minima, which denote dissonance. (Bottom row) auditory nerve (left) and brainstem (middle) responses similarly predict behavioral chordal sonority ratings (right) for the four most common triads in Western music. Chords considered consonant according to music theory (i.e., major, minor) elicit more robust subcortical responses and show an ordering expected by music practice (i.e., major > minor ≫ diminished > augmented). AN data from Bidelman and Heinz (2011); interval data from Bidelman and Krishnan (2009); chord data from Bidelman and Krishnan (2011).

To date, overwhelming evidence suggests that *cortical* integrity is necessary to support the cognitive aspects of musical pitch (Johnsrude et al., 2000; Ayotte et al., 2002; Janata et al., 2002; Peretz et al., 2009; Itoh et al., 2010). Yet, aggregating our findings from AN, human brainstem responses, and behavior provides a coherent picture of the emergence and time-course of musical pitch percepts in the ascending auditory pathway (Figure 5). Collectively, our findings demonstrate that the perceptual sonority and behavioral preference for both musical intervals and chords (triads) is well-predicted from early subcortical brain activity. Most notably, they also suggest that nascent neural representations relevant to the perception and appreciation of music are emergent well before cortical involvement at pre-attentive stages of audition.

As in language (Hickok and Poeppel, 2004), brain networks engaged during music likely involve a series of computations applied to the neural representation at different stages of processing. It is likely that higher-level abstract representations of musical pitch structure are first initiated in acoustics (Gill and Purves, 2009; McDermott et al., 2010). Physical periodicity is then transformed to musically relevant neural periodicity very early along the auditory pathway (AN; Tramo et al., 2001; Bidelman and Heinz, 2011), transmitted, and further processed (or at least maintained) in subsequently higher levels in the auditory brainstem (McKinney et al., 2001; Bidelman and Krishnan, 2009, 2011; Lee et al., 2009). Eventually, this information ultimately feeds the complex cortical architecture responsible for generating (Fishman et al., 2001) and controlling (Dowling and Harwood, 1986) musical percepts.

Importantly, it seems that even the non-musician brain is especially sensitive to the pitch relationships found in music and is enhanced when processing consonant relative to dissonant chords/intervals. The preferential encoding of consonance might be attributable to the fact that it generates more robust and synchronous phase-locking than dissonant pitch intervals. A higher neural synchrony for the former is consistent with previous neuronal recordings in AN (Tramo et al., 2001), midbrain (McKinney et al., 2001), and cortex (Fishman et al., 2001) of animal models which show more robust temporal responses for consonant musical units. For these pitch relationships, neuronal firing occurs at precise, harmonically related pitch periods; dissonant relations on the other hand produce multiple, more irregular neural periodicities. Pitch encoding mechanisms likely exploit simple periodic (cf. consonant) information more effectively than aperiodic (cf. dissonant) information (Rhode, 1995; Langner, 1997; Ebeling, 2008), as the former is likely to be more compatible with pitch extraction templates and provides a more robust, unambiguous cue for pitch (McDermott and Oxenham, 2008). In a sense, dissonance may challenge the auditory system in ways that simple consonance does not. It is conceivable that consonant music relationships may ultimately reduce computational load and/or require fewer brain resources to process than their dissonant counterparts due to the more coherent, synchronous neural activity they evoke (Burns, 1999, p. 243).

One important issue concerning the aforementioned FFR studies is the degree to which responses reflect the output of a *subcortical*, brainstem “pitch processer” or rather, a reflection of the representations propagated from more peripheral sites (e.g., AN). Indeed, IC architecture [orthogonal frequency-periodicity maps (Langner, 2004; Baumann et al., 2011), frequency lamina (Braun, 1999)] and response properties (critical bands, spectral integration) make it ideally suited for the extraction of pitch-relevant information (Langner, 1997). Yet, stark similarity between correlates observed in the AN (Bidelman and Heinz, 2011) and human brainstem FFRs (Bidelman and Krishnan, 2009, 2011) implies that the neurophysiological underpinnings of consonance and dissonance which may be established initially in the periphery, are no more than mirrored in brainstem responses observed upstream. Moreover, recent work also suggests that while brainstem responses may reflect pitch bearing-information, they themselves may not contain an adequate code to support all the intricacies of complex pitch perception (Gockel et al., 2011; but see Greenberg et al., 1987). Gockel et al. (2011), for instance, measured FFRs to complex tones where harmonics 2 and 4 were presented to one ear and harmonic 3 to the other (dichotic condition). Results showed that the FFR magnitude spectra under the dichotic listening condition were qualitatively similar to the sum of the response spectra for each ear when presented monaurally and furthermore, an absence of energy at F0 in the dichotic condition. These results imply that the FFR may preserve monaural pitch cues but may not reflect any additional “pitch” processing over and above what is contained in the combined representations from the periphery (i.e., AN). On the contrary, other studies have observed binaural interactions^1^ (Hink et al., 1980; Krishnan and McDaniel, 1998) and neural correlates for complex pitch attributes, e.g., “missing fundamental” (Galbraith, 1994), in the human FFR which are not observed in far-field responses generated from more peripheral auditory structures. These discrepancies highlight the need for further work to disentangle the potential differential (or similar) roles of brainstem and peripheral auditory structures in the neurocomputations supporting pitch. One avenue of investigation which may offer insight to these questions is to examine the degree to which neural plasticity – induced via training or experience – might differentially tune the neural encoding of pitch across various levels of the auditory pathway. Differential plasticity across levels might indicate different functional roles at different stages of auditory processing.

## Subcortical Plasticity in Musical Pitch Processing

The aforementioned studies demonstrate a critical link between sensory coding and the perceptual qualities of musical pitch which are independent of musical training and long-term enculturation. Electrophysiological studies thus largely converge with behavioral work, demonstrating that both musicians and non-musicians show both a similar bias for consonance and a hierarchical hearing of the pitch combinations in music (Roberts, 1986; McDermott et al., 2010). Yet, realizing the profound impact of musical experience on the auditory brain, recent studies have begun to examine how musicianship might impact the processing and perceptual organization of consonance, dissonance, and scale pitch hierarchy. Examining training-induced effects also provides a means to examine the roles of nature and nurture on the encoding of musical pitch as well as the influence of auditory experience on music processing.

### Neuroplastic effects on pitch processing resulting from musical training

**Figure 6:**
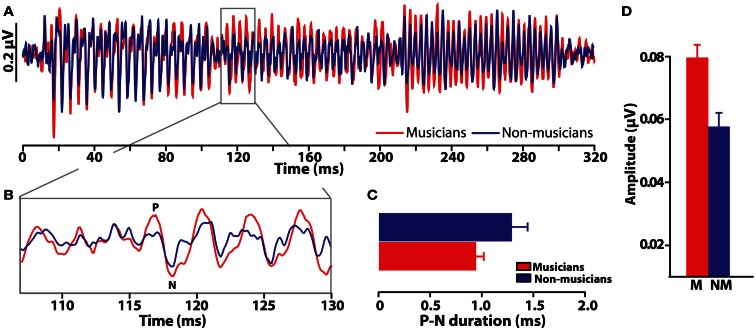
**Experience-dependent enhancement of brainstem responses resulting from musical training**. **(A)** Brainstem FFR time-waveforms elicited by a chordal arpeggio (i.e., three consecutive tones) recorded in musician and non-musicians listeners (red and blue, respectively). **(B)** Expanded time window around the onset response to the chordal third (≈117 ms), the defining note of the arpeggio sequence. Relative to non-musicians, musician responses are both larger and more temporally precise as evident by their shorter duration P-N onset complex **(C)** and more robust amplitude **(D)**. Musical training thus improves both the precision and magnitude of time-locked neural activity to musical pitch. Error bars = SEM. Data from Bidelman et al. (2011d).

Comparisons between musicians and non-musicians reveal enhanced brainstem encoding of pitch-relevant information in trained individuals (Figure 6) (Musacchia et al., 2007; Bidelman and Krishnan, 2010; Bidelman et al., 2011a,d). Additionally, as indicated by shorter, less “jittered” response latencies, musicians’ neural activity is also more temporally precise than that of non-musicians. Musical training therefore not only magnifies the “gain” of subcortical brain activity (Figure 6D) but also refines it by increasing the temporal precision of the brain’s response to complex pitch (Figure 6C) (Bidelman et al., 2011d). Interestingly, these neural indices are correlated with an individual’s degree of musical training/experience (Musacchia et al., 2007; Wong et al., 2007) as well as their perceptual abilities (Bidelman et al., 2011b, 2013). Together, these enhancements observed in musicians’ brainstem FFRs indicate that experience-dependent plasticity, well-established at cortical levels of processing, also extends to *subcortical* levels of the human brain. A natural question which then arises is the degree to which musical training might modulate the inherent (subcortical) auditory processing subserving musical consonance-dissonance reviewed earlier.

### Experience-dependent changes in the psychophysiological processing of musical consonance

At a subcortical level, recent studies have demonstrated more robust and coherent brainstem responses to consonant and dissonant intervals in musically trained listeners relative to their non-musician peers (Lee et al., 2009). Brainstem phase-locking to the temporal periodicity of the stimulus envelope – a prominent correlate of roughness/beating (Terhardt, 1974) – is also stronger and more precise in musically trained listeners (Lee et al., 2009). These results suggest that brainstem auditory processing is shaped experientially so as to refine neural representations of musical pitch in a behaviorally relevant manner (for parallel effects in language, see Bidelman et al., 2011a). They also indicate that subcortical structures provide differential processing of musical pitch above and beyond “innate” representations which might be established in the periphery (Tramo et al., 2001; Bidelman and Heinz, 2011).

Recent work also reveals similar experience-dependent effects at a cortical level. Consonant chords, for example, elicit differential hemodynamic responses in inferior and middle frontal gyri compared to dissonant chords regardless of an individual’s musical experience (Minati et al., 2009). Yet, the hemispheric laterality of this activation differs between groups; while right lateralized for non-musicians, activation is more symmetric in musicians suggesting that musical expertise recruits a more distributed neural network for music processing. Cortical brain potentials corroborate fMRI findings. Studies generally show that consonant and dissonant pitch intervals elicit similar modulations in the early components of the ERPs (P1/N1) for both musicians and non-musicians alike. But, distinct variation in the later waves (N2) are found nearly exclusively in musically trained listeners (Regnault et al., 2001; Itoh et al., 2003, 2010; Schön et al., 2005; Minati et al., 2009). Thus, musicianship might have a differential effect on the time-course of cortical auditory processing; musical training might exert more neuroplastic effects on later, endogenous mechanisms (i.e., N2) than on earlier, exogenous processing (e.g., P1, N1). Indeed, variations in N2 – which covaries with perceived consonance – are exaggerated in musicians (Itoh et al., 2010). These neurophysiological findings are consistent with recent behavioral reports which demonstrate musicians’ higher sensitivity and perceptual differentiation of consonant and dissonant pitches (McDermott et al., 2010; Bidelman et al., 2011b,d). Recently, McDermott et al. (2010) have observed a correspondence between a listener’s years of musical training and their perceptual sensitivity for harmonicity (but not roughness) of sound. Thus, it is possible that musician’s higher behavioral and neurophysiological propensity for musical consonance might result from an experience-dependent refinement in the internalized templates for complex harmonic sounds. Taken together, neuroimaging work indicates that while sensory consonance is coded in both musically trained and untrained listeners, its underlying neural representations can be amplified by musical expertise. In a sense, whatever aspects of musical pitch are governed by innate processing, musical experience can provide an override and exaggerate these brain mechanisms.

Limitations of these reports are worth mentioning. Most studies examining the effects of musical training on auditory abilities have employed cross-sectional and correlational designs. Such work has suggested that the degree of a musicians’ auditory perceptual and neurophysiological enhancements is often positively associated with the number of years of his/her musical training and negatively associated with the age at which training initiated (e.g., Bidelman et al., 2013; Zendel and Alain, 2013). These types of correspondences hint that musicians’ auditory enhancements might result from neuroplastic effects that are modulated by the amount of musical exposure. It should be noted however, that comparisons between highly proficient musicians and their age-matched non-musician peers offers an imperfect comparison to address questions regarding the role of *experience* on brain and behavioral processing; causality cannot be inferred from these quasi-experimental, cross-sectional designs. To truly gauge the role of musical experience on harmonicity, consonance perception, and brainstem pitch processing, longitudinal experiments with random subject assignment are needed (e.g., Hyde et al., 2009; Moreno et al., 2009). Interestingly, recent training studies with random subject assignment suggests that even short-term auditory training (∼1 month) can positively alter brainstem function as indexed via the FFR (Carcagno and Plack, 2011). Presumably, the high intensity and duration of long-term musical training would only act to amplify these plastic effects observed in the short-term supporting the notion that experience and “nurture” drive the aforementioned plasticity. Future work may also look to developmental studies (e.g., Schellenberg and Trainor, 1996; Trainor et al., 2002) to disentangle the contributions of experiential and innate factors in musical pitch processing.

## Is There a Neurobiological Basis for Musical Pitch?

There are notable commonalities (i.e., universals) among many of the music systems of the world including the division of the octave into specific scale steps and the use of a stable reference pitch to establish key structure. In fact, it has been argued that culturally specific music is simply an elaboration of only a few universal traits (Carterette and Kendall, 1999), one of which is the preference for consonance (Fritz et al., 2009). Together, our recent findings from human brainstem recordings (Bidelman and Krishnan, 2009, 2011) and single-unit responses from the AN (Bidelman and Heinz, 2011) imply that the perceptual attributes related to such preferences may be a byproduct of innate sensory-level processing. These results converge with previous behavioral studies with infants which have shown that months into life, newborns prefer listening to consonant rather than dissonant musical sequences (Trainor et al., 2002) and tonal rather than atonal melodies (Trehub et al., 1990). Given that these neurophysiological and behavioral effects are observed in the absence of long-term enculturation, exposure, or music training, it is conceivable that the perception of musical pitch structure develops from domain-general processing governed by the fundamental capabilities of the auditory system (Tramo et al., 2001; McDermott and Hauser, 2005; Zatorre and McGill, 2005; Trehub and Hannon, 2006; Trainor, 2008).

It is interesting to note that musical intervals and chords deemed more pleasant sounding by listeners are also more prevalent in tonal composition (Budge, 1943; Vos and Troost, 1989; Huron, 1991; Eberlein, 1994). A neurobiological predisposition for simpler, consonant intervals/chords – as suggested by our recent studies – may be one reason why such pitch combinations have been favored by composers and listeners for centuries (Burns, 1999). Indeed, the very arrangement of musical notes into a hierarchical structure may be a consequence of the fact that certain pitch combinations strike a deep chord with the architecture of the nervous system.

## Conclusion

Brainstem evoked potentials and AN responses reveal robust correlates of musical pitch at subcortical levels of auditory processing. Interestingly, the ordering of musical intervals/chords according to the magnitude of their subcortical representations tightly parallels their hierarchical arrangement as described by Western music practice. Thus, information relevant to musical consonance, dissonance, and scale pitch structure emerge well before cortical and attentional engagement. The close correspondence between subcortical brain representations and behavioral consonance rankings suggests that listeners’ judgments of pleasant- or unpleasant-sounding pitch relationships may, at least in part, be rooted in early, pre-attentive stages of the auditory system. Of the potential correlates of musical consonance described throughout history (e.g., acoustical ratios, cochlear roughness/beating, neural synchronicity), results suggest that the *harmonicity of neural activity* best predicts human judgments. Although enhanced with musical experience, these facets of musical pitch are encoded in non-musicians (and even non-human animals), implying that certain fundamental attributes of music listening exist in the absence of training, long-term enculturation, and memory/cognitive capacity. It is possible that the preponderance of consonant pitch relationships and choice of intervals, chords, and tuning used in modern compositional practice may have matured based on the general processing and constraints of the sensory auditory system.

## Conflict of Interest Statement

The authors declare that the research was conducted in the absence of any commercial or financial relationships that could be construed as a potential conflict of interest.

## Footnotes

^1^A binaural interaction component (BIC) is derived from scalp-recorded ERPs as the difference between potentials evoked via binaural stimulation from the summed responses evoked by monaural stimulation. Assuming confounding factors such as acoustic cross-talk and middle ear reflex are eliminated, the resulting BIC response is thought to reflect neural interaction in the outputs from both ears converging at or above the level of the brainstem (Krishnan and McDaniel, 1998). Binaural interaction has been observed in brainstem, middle-latency, and cortical auditory evoked potentials and can be used to investigate the central interaction of auditory information (McPherson and Starr, 1993).
